# On the Role of the Blood Vessel Endothelial Microvilli in the Blood Flow in Small Capillaries

**DOI:** 10.1155/2015/529746

**Published:** 2015-10-28

**Authors:** Vladimir Makarov, Lidia Zueva, Priscila Sanabria, William Dave Wessinger, Tatiana Golubeva, Igor Khmelinskii, Mikhail Inyushin

**Affiliations:** ^1^Department of Physics, University of Puerto Rico (UPR), San Juan, PR 00931, USA; ^2^Department of Neuroscience, Central University of Caribbean (UCC), Bayamon, PR 00960, USA; ^3^Department of Physiology, Central University of Caribbean (UCC), Bayamon, PR 00960, USA; ^4^Department of Pharmacology and Toxicology, University of Arkansas for Medical Sciences (UAMS), Little Rock, AR 72205, USA; ^5^Department of Vertebrates Zoology, Lomonosov Moscow State University (MSU), Moscow 119234, Russia; ^6^Faculty of Sciences and Technology, University of Algarve (UAlg), 8005-139 Faro, Portugal

## Abstract

Endothelial microvilli that protrude into the capillary lumen, although invisible in the optical microscopy, may play an important role in the blood flow control in the capillaries. Because of the plug effects, the width of the gap between the capillary wall and the blood cell is especially critical for the blood flow dynamics in capillaries, while microvilli located on the capillary wall can easily control the velocity of the blood flow. We report that microvilli in the capillaries of different vertebrate species have similar characteristics and density, suggesting similarities between the respective regulation mechanisms. A simplified physical model of the capillary effective diameter control by the microvilli is presented.

## 1. Introduction

The diameter of the blood vessel capillaries in most vertebrates is about 25% smaller than the mean diameter of the erythrocytes, causing blood cell deformation during the capillary flow [1, 2]. Thus, the blood flow in the capillaries is that of a series of the red blood cells, separated by the plasma trapped between the two neighboring cells, termed the bolus blood flow or flow with the plug effect [3, 4]. A model of the idealized motion of blood in capillary blood vessels, including a series of flat disks representing erythrocytes separated by plasma, has been analyzed earlier [4]. This study revealed a significant difference between the mean velocity of the plasma and that of the red blood cells, dependent on the gap between the red blood cells and the capillary wall. This clearly shows that one should not use the measured velocity of the red blood cells as the mean blood flow velocity in the capillary; this latter value is dependent on the cell-wall gap size [4]. The effect of the cell-wall gap on the flow velocity depends on the structure of the endothelial wall; for example, the flow is affected by the presence of the porous endothelial layer [5]. The idea that the gap between the erythrocyte and the endothelial wall may itself be controlled has not been considered at that time.

It is generally recognized that the capillary blood flow is tightly regulated [6, 7]. Usually, the regulation of the blood flow in small blood vessels is associated with the contractile elements outside the endothelium, like the external muscle walls of the arterioles or the external contractile glial cells (pericytes) on the venules and capillaries [6, 8]. Here we suggest an additional mechanism of the blood flow regulation by the specialized endothelial microvilli, presenting a simplified model of how the microvilli could contribute to controlling the flow in small capillaries.

The specialized endothelial microvilli protruding into the blood vessel lumen, invisible in the optical microscopy, were discovered soon after the electron microscope had been introduced into the practice [9, 10]. These hair-like processes are about 0.1 *μ*m in diameter and 0.5–3 *μ*m long [10]. The density of the microvilli on the endothelial cell surface increases during ischemia and in hypertensive animals [11–14]. On the other hand, application of NO reduces the count and length of the endothelial microvilli [15]. The microvilli formation may enhance thrombosis by promoting platelet aggregation [16] or aggregate and promote internalization of virulent bacteria into the vascular endothelial cells [17]. The microvilli are associated to the intermediate filament cytoskeleton, being very rich in ezrin and moesin, two members of the ERM (ezrin/radixin/moesin) family, and their respective transmembrane-binding proteins, such as CD44 with actin polymerization, all colocalized within these membrane protrusions [17]. All of these data suggest that microvilli are highly dynamic structures; they can appear or disappear or change their length upon regulation. For example, it was shown that ischemia may reduce the effective capillary diameter by a factor of two from its normal value, possibly because of the microvilli “swelling” [18]. Our electron-microscopy study in mammals (rats and mice) revealed that the endothelial microvilli may participate in the plasma flow regulation by controlling the erythrocyte plug effect. We also found that similar endothelial microvilli are also present in birds, suggesting that the microvilli and their associated mechanisms of regulation may be present in all vertebrates. These data led us to the hypothesis that the endothelial microvilli are an important player in the blood-tissue exchange, as they can control the blood cell speed and thus the plasma flow speed in the cell-wall gap.

Here we present an electron microscopy study of the microvilli in small capillaries in mammals and birds, along with a simplified model of the control of the cell-wall gap between the erythrocytes and the endothelial wall of the capillary, and thus of the blood flow in the capillaries.

## 2. Methods

### 2.1. Animals and Section Preparation

All of the experimental procedures were performed in accordance with the US Public Health Service Publication Guide for the Care and Use of Laboratory Animals and were approved by the Animal Care and Use Committee at Universidad Central del Caribe. Cortical samples from Sprague-Dawley rats and from B6 mice of either sex of 150–200 days of age were used. Bird blood vessels were studied using the sections obtained from our previous work on the pied flycatcher [19].

We used the standard staining method for the electron microscopy as described previously [19]. Briefly, samples were fixed in the mixture of 2.5% glutaraldehyde with 4% paraformaldehyde in 90 mM sodium cacodylate buffer with 0.02 mM CaCl_2_ added and pH adjusted to 7.2–7.4. Samples were kept in the fixative at 5°C for 24 hours. The samples were (1) washed for 20 min in 90 mM sodium cacodylate buffer, (2) postfixed for 30 min in 1% osmium tetroxide (OsO_4_) with a few granules of potassium ferrocyanide (K_4_Fe(CN)_6_·3H_2_O), (3) incubated for 30 min in 1% osmium tetroxide, and (4) incubated in 2% aqueous uranyl acetate (UO_2_(CH_3_OCO)·H_2_O) for 1 hour. The samples were washed for 20 min in distilled water after each of the steps (1)–(4). The samples were then dehydrated in a graded acetone series and embedded in a 1 : 1 mixture of EMBed-812 and SPURR (EM Sciences). Ultrathin sections (50–60 nm) were cut with a Leica Ultracut Ultramicrotome, mounted on formvar-coated copper slot grids and examined with a JEM 100CX II transmission electron microscope (JEOL). The chemicals for buffers and solutions were purchased from Sigma-Aldrich (MO, USA), and the stains for the electron microscopy were purchased from EM Sciences (PA, USA).

## 3. Results

### 3.1. Microvilli in the Capillaries of Mammals and Birds

**Figure 1:**
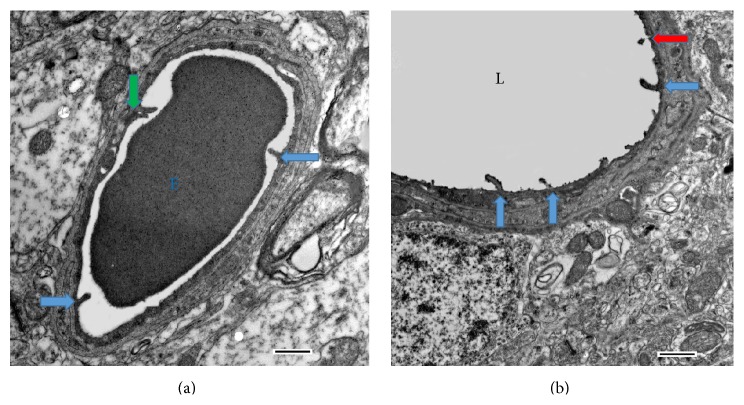
The microvilli in the rat capillary: electron microphotography. (a) An erythrocyte (E) inside the capillary; the microvilli (arrows) produce visible invaginations on the erythrocyte wall. (b) A larger blood vessel. L: the lumen of the vessel. The microvilli are marked with arrows; green arrow: double microvilli (see the text); red arrow: a radially cut microvillus, with axial symmetry. Scale bar: 500 nm.

The transmission electron microscopy of the rat brain cortical capillaries showed a large number of microvilli on the luminal surface of the endothelial cells. They were 0.1 to 0.5 *μ*m long, with the diameter of about 0.1 *μ*m. The erythrocytes in the capillary were frequently found surrounded by the microvilli; especially in the zones of bifurcation there the microvilli density is usually elevated, probably allowing for a better control of the blood flow distribution between the two vessels. The rigidity of the microvilli seemed high enough to induce invaginations in the erythrocyte membrane (Figure 1(a)). The linear density of the microvilli in the capillaries varied from 0.1–0.2 to 1-2 per *μ*m of the capillary wall cross section length in normal animals without any treatment, both on longitudinal and radial cross sections of the blood vessels, discarding those microvilli fragments that appeared unattached to the wall. Similar microvilli were present in larger vessels as well, at an even larger density (Figure 1(b)). Some of the bent microvilli were found cut, showing their axial symmetry (Figure 1(b), red arrow).

**Figure 2:**
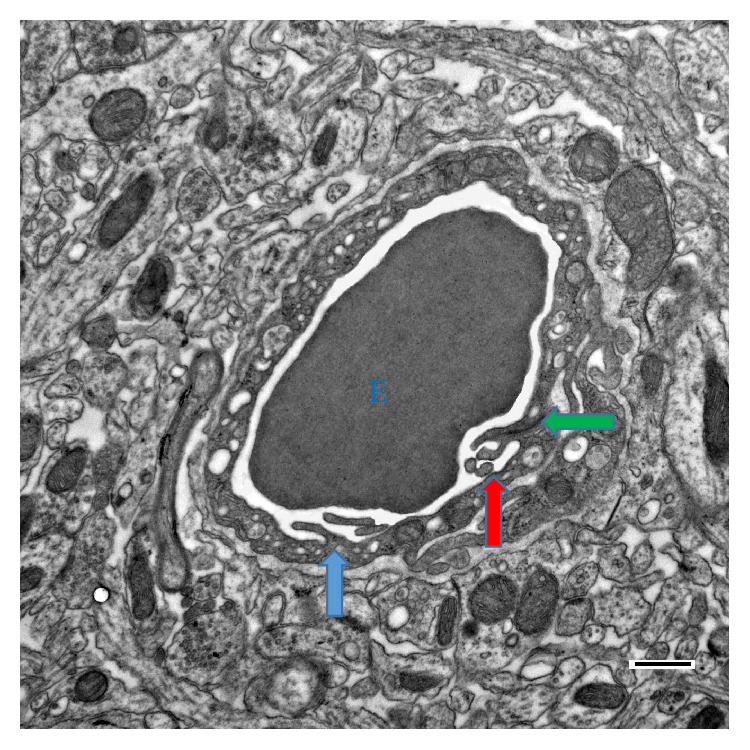
The microvilli in the mouse capillary: electron microphotography. An erythrocyte (E) inside the capillary; the microvilli (arrows) produce visible invaginations on the erythrocyte wall. Green arrow: double microvilli (see the text); red arrow: radially cut microvilli, apparently with axial symmetry. Scale bar: 500 nm.

Very similar microvilli appear on the luminal surface of the endothelium in mice capillaries (Figure 2); some of them were cut transversally, some radially, also showing their axial symmetry (Figure 2, the red arrow). Double microvilli may be formed on junctions of neighboring endothelial cells, appearing quite frequently in mice and rats (green arrows in Figures 1 and 2).

**Figure 3:**
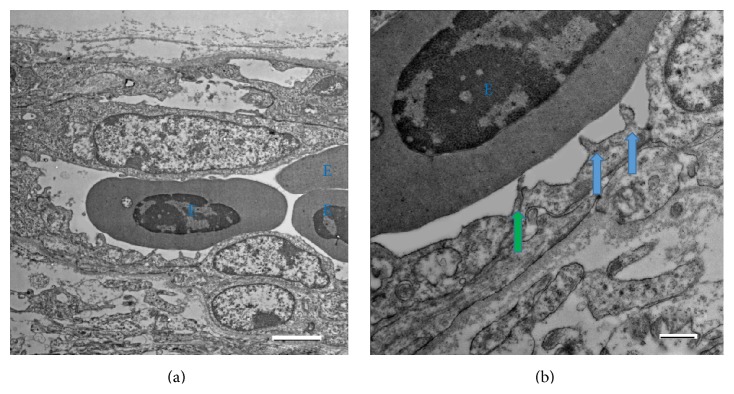
Electron microphotography of the microvilli in a bird capillary. (a) Erythrocytes (E) in the longitudinally cut bird capillary. (b) The same photo at a larger magnification. The microvilli are marked with arrows; green arrow: double microvilli. Scale bar on (a): 2 *μ*m, on (b): 500 nm.

Motivated by the results obtained in the rat and mouse capillaries, we reexamined the samples from our previous work on birds [19], where the sections of the capillaries revealed very similar microvilli in their lumen. Similarly to the mammals, double microvilli were frequently detected on the junctions of the neighboring endothelial cells (Figure 3, green arrow).

The discovery of similar microvilli in the lumen of the capillaries of the two different vertebrate classes suggests that these structures represent an ancient mechanism, necessary for the proper functioning of the blood vessels. We therefore suggest that the length and density of the microvilli may be important for the blood flow dynamics and regulation, affecting the blood-tissue material exchange rates and efficiency.

### 3.2. A Physical Model of the Blood Flow Control in the Capillaries by the Endothelial Microvilli

A detailed analysis of the theoretical models describing the flow dynamics of the heterogeneous fluids (liquid phase with suspended cells of different shape) in the capillary systems has been presented earlier (Secomb et al.) [3, 4, 20, 21]. Lew and Fung, 1970 [4], demonstrated that, representing the cells (erythrocytes) by flat disks, the average velocity of the liquid phase may be significantly different from that of the disks, provided the disk diameter is close to the capillary diameter. In such cases, the ratio of these two velocities is dependent on the ratio of the capillary diameter to the cell diameter. Note that the following boundary conditions were used in all of these models: (i) the velocity of the liquid at the capillary wall is zero; (ii) the velocity of the liquid at the cell surface equals the cell velocity. The model calculations demonstrated that the blood plasma layer near the capillary wall moves at the velocity that strongly depends on the viscosity, while the more centrally located plasma trapped between the consecutive cells moves at a different velocity, equal to that of the suspended cells.

These conclusions were confirmed in the experiments showing that the red blood cell velocity is different from that of the dye added to the plasma [22, 23]. Taking into account that the already mentioned microvilli may change their density, shape, and length depending on conditions, therefore affecting the effective diameter of the capillary as regards the erythrocyte motion, we propose that microvilli may control not only the capillary blood flow resistance, but also the ratio between the erythrocyte velocity and the blood plasma velocity. The control of this ratio will affect the material exchange between the blood and the capillary endothelium.

**Figure 4:**
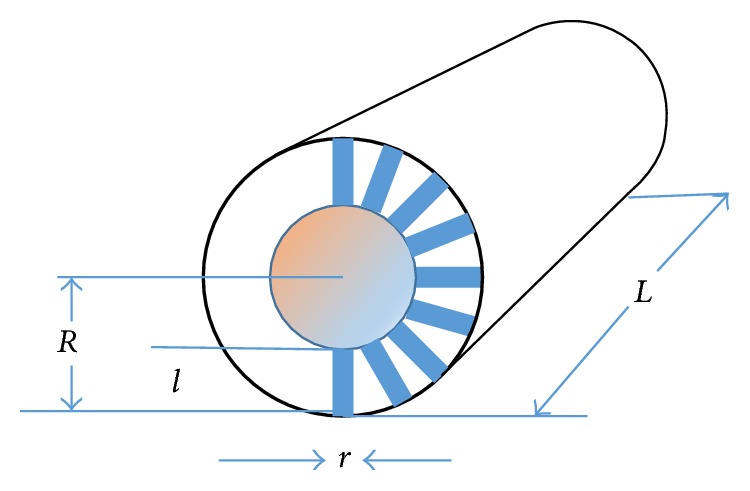
The physical model: the capillary is represented by a cylinder of the length *L* and radius *R*, each of the microvilli is a cylinder of the length *l* and the diameter *r*.

Presently we propose a simplified phenomenological model of the effective capillary diameter regulation by the endothelial microvilli, in function of their diameter, length, and number density (see Figure 4).

We shall use the following notations: *r* is the microvilli radius, *l* their length, *R* the capillary radius, and *L* its length. Thus, we can estimate the critical count of the microvilli, corresponding to the capillary radius reduced from *R* to *R* − *l*. The cross section area of each microvillus is given by

(1) $$\begin{array}{c} S=\pi r^{2}. \end{array}$$

The area of the internal capillary surface with the radius *R* − *l* is given by

(2) $$\begin{array}{c} S_{0}=2\pi L\left(\;R-l\;\right). \end{array}$$

The critical count of microvilli is thus given by

(3) $$\begin{array}{c} n_{c}=\frac{2L\left(R-l\right)}{r^{2}}. \end{array}$$

Thus, the critical density of microvilli per unit area is given by

(4) $$\begin{array}{c} \rho_{c}=\frac{n_{c}}{2\pi RL}=\frac{\left(R-l\right)}{\pi Rr^{2}}. \end{array}$$

We define *ρ*_*c*_ as the critical density, corresponding to the microvilli so dense that they form a new surface inside the vessel walls (Figure 4). Usually the density may be lower; therefore, we shall define the effective radius *r*_eff_ of the capillary dependent on the microvilli density *ρ* and their other parameters as follows:

(5) $$\begin{array}{c} r_{eff}=\left(\;R-l\;\right)R\frac{\rho_{c}}{\left(R-l\right)\rho_{c}+l\rho }. \end{array}$$

It follows from (5) that we obtain *r*_eff_ = *R* at *ρ* = 0, and *r*_eff_ = (*R* − *l*) at *ρ* = *ρ*_*c*_, as expected. According to Poiseuille's law, the volume of a homogeneous liquid passing through a cylindrical pipe per unit time is expressed by

(6) $$\begin{array}{c} Q\left(\;r\;\right)=\frac{\pi R^{4}}{8\eta lF}\Delta p. \end{array}$$

We should substitute *r*_eff_ for *R*, with Δ*p* being the pressure difference between the inlet and outlet sections of the pipe, *η* the fluid viscosity, and *F* the pipe length. It follows from (5)-(6) that the amount of fluid depends on *r*_eff_^4^. Therefore, small changes in *r*_eff_ may significantly affect the amount of fluid passing through the capillary. For a homogeneous fluid in a cylindrical pipe, the fluid velocity distribution is described by a parabolic function, with zero velocity at the pipe wall and the maximum velocity on the pipe axis. However, blood is heterogeneous; therefore, the regulation of the flow in the gap between the capillary wall and the red blood cells may be even more efficient. Indeed, Lew and Fung [4] analyzed blood flow in the capillary with small cell-wall gap (plug flow). They found that there is a significant difference between the mean plasma velocity and the mean blood cell velocity, with the ratio of the two dependent on the ratio of the capillary diameter to the cell diameter. Taking into account (5) and the results reported earlier [4], we obtained an approximate relationship for the ratio of the average velocities of the erythrocytes and of the blood plasma in function of the effective capillary radius and the erythrocyte radius *r*_er_, representing erythrocytes by thin rigid disks:

(7) $$\begin{array}{c} \frac{{\overset{-}{\upsilon }}_{er}}{{\overset{-}{\upsilon }}_{p}}=\mu ={\left(\;\frac{r_{eff}-nr_{er}}{r_{eff}}\;\right)}^{m+1}. \end{array}$$

To obtain (7), we assumed that the friction coefficient *γ* for the erythrocyte motion inside the capillary is given by

(8) $$\begin{array}{c} \gamma =\eta {\left(\;\frac{nr_{er}}{r_{eff}}\;\right)}^{m}. \end{array}$$

Here, *n* and *m* are phenomenological parameters, both varying between zero and unity, and *γ* is proportional to the plasma viscosity. It follows from (7) that the velocity ratio is zero for *r*_eff_ = *nr*_er_; thus the erythrocytes are not moving; the case of *r*_eff_ < *nr*_er_ has no physical sense; while, for *r*_eff_ ≫ *nr*_er_, we obtain *μ* ≈ 1, as reasonably expected. Thus, (5) provides a link between the microvilli structure and density and the blood flow regulation in a capillary.

Since the microvilli structure and density may be altered by the regulation mechanisms, our model of the effective capillary radius controlled by the microvilli length, radius, and density allows for an efficient control of the blood flow dynamics in the capillaries.

## 4. Discussion

We report that the inner surface of the endothelial cells in the capillaries has the same microvillar structure (Figures 1 and 2) that has been previously described in larger blood vessels [9–12, 15, 17, 18]. We also report that the same endothelial microvilli are found in bird capillaries as well (Figure 3), extending their presence to various classes of vertebrates. According to the present study, the linear density of the microvilli on the capillary wall cross section varied between *ρ*_lin_ = (0.1,…, 2) *μ*m^−1^ in rats without any special treatment. We estimated the density of the microvilli per unit area *ρ* using the following formula:

(9) $$\begin{array}{c} \rho =\frac{\rho_{lin}}{h\left(d/h+1\right)}. \end{array}$$

Here, *h* = 0.05 *μ*m is the cut thickness and *d* = 0.1 *μ*m the approximate microvillus diameter. The calculation produced *ρ* = (0.7,…, 13) *μ*m^−2^ for the microvilli density in capillaries, which is much lower than their density in large arteries [10] but still sufficiently high to affect the blood flow in the capillaries. The density of the microvilli on the endothelial cell surface increases during ischemia and in hypertensive animals [11–13].

Microvilli are associated to the intermediate filament cytoskeleton, being very rich in ERM proteins that usually crosslink the actin filaments with the plasma membranes [17]. This suggests that the microvilli length and other characteristics may be controlled by the pO_2_/pCO_2_ and other metabolic regulators. Indeed, ischemia may reduce the effective capillary diameter by the factor of two from its normal value, possibly because of the microvilli “swelling” [18]. Since the microvilli structure (length and radius) and density may be changed depending on conditions, the presently proposed simple model of the effective capillary radius control by changing the microvilli length, radius, and density describes an efficient way of the blood flow regulation in capillaries (Figure 4). The proposed mechanism of the blood flow regulation by the microvilli is only a hypothesis, lacking sufficient experimental evidence. Further advancement of this research will target the development of the experimental methods producing evidence for the proposed mechanism.

On the other hand, the red blood cells tend to plug the capillaries preventing the plasma from flowing freely as it does in large blood vessels [4]. While the red blood cells move fairly steadily, the plasma trapped between the cells and the capillary wall experiences turbulent vortices and recirculation zones, with increased hydrodynamic resistance to the blood flow. As it was shown theoretically, there is a significant difference between the mean velocity of plasma and that of the red blood cells in capillaries, controlled by the cell-wall gap [4]. These ideas found experimental confirmation [22, 23]; therefore, the control of the plasma flow through the cell-wall gap by the microvilli located on the capillary wall may make the control of the capillary blood flow more efficient. Note that the extra turbulence of the plasma should facilitate the material exchange between the plasma and the capillary wall. Thus, the control of the plug flow may affect the exchange itself. The microvilli also augment the contact surface of the endothelium cells with the plasma, possessing special receptors providing additional binding and internalization [16, 17, 24]. Still, the density of microvilli is not high enough to augment the contact surface significantly; also, the microvilli are too thin and long to promote efficient exchange through their surface. Also, the most dense population of microvilli is found in larger vessels, like the aortic arch with its thick walls limiting the exchange [10]. All of these data indicate some participation of the microvilli in the exchange processes, insufficiently understood and warranting additional theoretical and experimental studies of the exchange regulation by the microvilli.

## 5. Conclusions

1. Very similar endothelial microvilli were found in the lumen of capillaries in different species of mammals and birds.
2. A simplified physical model predicts that microvilli may efficiently regulate the blood flow in the capillaries.
